# Determinants of the Mobile Health Continuance Intention of Elders with Chronic Diseases: An Integrated Framework of ECM-ISC and UTAUT

**DOI:** 10.3390/ijerph19169980

**Published:** 2022-08-12

**Authors:** Xiu-Fu Tian, Run-Ze Wu

**Affiliations:** 1 College of Business, Jiaxing University, Jiaxing 314001, China; 2 College of Economics, Jiaxing University, Jiaxing 314001, China

**Keywords:** mobile health, elders with chronic diseases, continuance intention, ECM-ISC, UTAUT

## Abstract

With the deepening of population aging in China, chronic diseases are a major public health concern that threatens the life and health of nationals. Mobile health or mHealth can effectively monitor chronic diseases, which holds vital significance to the alleviation of social pressure caused by aging. To patients with chronic diseases, mHealth cannot give full play to its value, only when it is used in the long term. However, there is not yet research exploring mHealth continuance intention from the perspective of elders with chronic diseases. So, this research represents the first attempt to empirically analyze mHealth continuance intention from the perspective of elders with chronic diseases. The purpose of this research is to make up the research gap of the mHealth field and to put forward theoretical and practical implications based on research results. To obtain research data, a questionnaire was conducted. A total of 926 copies were collected online and 527 copies were collected offline. The structural equation model (SEM) was used for data analysis. Research results suggest that confirmation can significantly influence satisfaction, performance expectancy and effort expectancy. Meanwhile, confirmation and performance expectancy can significantly influence satisfaction. Additionally, effort expectancy, performance expectancy, social influence and facilitating conditions can directly and significantly influence continuance intention. Among them, performance expectancy can directly influence continuance intention in the most significant way. This research provides solid evidence for the validity of the integrated model of ECM-ISC and UTAUT in the mHealth field, which can be a theoretical basis for mHealth operators’ product R&D.

## 1. Introduction

Current human society is confronted with a great variety of problems, such as poverty and climate change, of which health is one of the most serious concerns going through the whole history of human civilization and consuming a large number of social resources. As a shared pursuit of all mankind, health can lay a solid foundation for the progress of social civilization. However, disease is something inevitable that will accompany a person all his life. With the gradual improvement of people’ s living standards, there has been a growing demand for medical and health services [1,2]. As estimated by the United Nations, the population aged above 60 years old will have increased to 454 million by the year 2050 in China, taking up 34% of the national total, which can be judged by standards of the World Health Organization as the deep population aging stage [3]. Additionally, according to the *2014 Report on Chinese Residents’ Chronic Disease and Nutrition*, there were around 845 Chinese diagnosed with chronic diseases in 2019, of which overweight people accounted for 50% of the total among grown-ups, and deaths resulted from chronic diseases took up 88.5% of the total. Among these deaths, 80.7% were caused by cerebrovascular diseases, cancers and chronic respiratory system diseases [4]. Moreover, following the deepening of population aging, there is a surge in medical demands and medical fees, and a serious shortage of medical resources. All these are serious social problems, which aggravate the contradiction between the increasing demand for public medical care and the tenseness of medical resources [5]. Therefore, to strengthen monitoring and the prevention of chronic diseases holds vital significance to the alleviation of social pressure brought about by population aging.

Driven by the sharpening of the public awareness of health, there is a growing demand for medical and health services, which is mainly reflected as actively requiring the monitoring of vital signs, prevention of chronic diseases and health intervention [6]. Pitifully, however, in China, medical resources are unproportionally distributed and badly needed; supply and demand of medical services are imbalanced, and some services are even wasted [7]. With a population that accounts for 22% of the world total, Chinese medical resources are otherwise lower than 2% of the world total, and 80% of the resources are concentrated in urban areas, leading to a scarcity of medical resources in villages and remote areas [8]. As population aging speeds up, the conflict between the growing medical demand and the scarcity and disproportional distribution of medical resources is a major obstacle to the development of China’s medical undertakings. How to effectively ensure different parts of China to have access to adequate medical resources remains a social issue of great significance [9].

Under the medical development background, a new-type medical style, known as mHealth (abbreviation for “Mobile Health”), has appeared. Propelled by the development of information technologies and government policy support, the development of mHealth has been an irresistible trend [10]. By changing the traditional medical models, mHealth can significantly improve the resource allocation efficiency, lower the social medical cost and improve people’s overall health level [11,12]. Digital technology is becoming an important contributor to promoting the spread of health services and the development of public health. Particularly, mobile wireless technology is playing an essential role in the delivery of medical services, relying on its ease of use, a wide coverage and a high efficiency [13,14,15]. mHealth can change and even reverse traditional medical diagnosis models, medical talent development and scientific research models. Online medical care makes intelligent self-diagnosis possible for users through mobile medical care means [16,17,18]. Additionally, through mHealth, patients can realize online communication with doctors to ensure timely adjustment of medication for improvement of treatment effects. This can not only address the disproportional distribution of medical resources, but also cope with the contradiction between the shortage of medical resource supply and people’s increasing health needs [19,20]. Therefore, development of mHealth can reduce waste of medical resources and fees by handling some of the consultants who do not need to seek medical advice offline. These consultants can also reduce their time cost of going to the hospital. In this way, the limited high-quality medical resources can be reserved for those that are in emergent conditions.

In spite of the sharp increase in China’s mHealth market, the low utilization and adoption rate of mHealth services by elders with a high medical care demand has impeded the development and popularization of mHealth. As revealed by the poll of the Pew Research Center in 2012, 31% of mobile phone users consulted about their health problems via their smartphones, but the adoption and utilization rate of mHealth services remained low among users aged above 65 years old and those whose educational degree was below the senior high school: mobile users aged 18 to 49 were more likely to acquire health information through their mobile phones [21]. On the other hand, the American government promulgated regulations on the effective use of the electronic health record, requiring every clinic to enable more than half of its patients to have access to their health information and communicate with doctors online so as to improve the diagnosis quality and bring down medical fees [22]. In China, the largest mHealth company, Chunyuyisheng.com, has more than 33 million registered users, but active users take up around 5.0% [23]. Addition, the research of Levy [24] indicated that health applications have a high deletion rate, meaning that application users’ intention of continuance usage is lacking. 

Based on the above discussions, old users’ low adoption and utilization rate has been a main hindrance to mHealth’s sustainable development. Different from the youth and the middle-aged, elders might reject innovative products because of lacking skills to learn something new. On the other hand, though the demand of mHealth services is constantly increasing, digital health information systems around the world have are all confronted with a major challenge; namely users’ continuance usage. Research of the information system field sheds light on the fact that continuance usage is a main characteristic of users’ steady user behavior, which is also a critical standard to measure whether the system is successful or not [25]. Nevertheless, the extant literature related to mHealth are concerned mostly about technological feasibility, clinical effects, initial adoption of the information system, etc. Little attention is paid to the behavioral intention of users after adopting mHealth services. Thereby, in this research field remains a research gap crying for more research attempts at the practice level and the theoretical level. Only when more research attention is paid to this field can mHealth theories be further enriched and more effective marketing strategies be drawn up for mHealth enterprises. Thus, this research includes ECM-ISC, a relatively authoritative model in the field of information system continuance intention, into mHealth research. In fact, ECM-ISC is also verified by research of different fields. For example, Kim et al. [26] investigated users’ mobile accommodation-related continuance intention based on the ECM. Results suggested that the expectation confirmation has a significant influence on users’ perceived usefulness and satisfaction and that satisfaction had the greatest influence on users’ continuance use intention. Moreover, Cheng [27] adopted ECM to verify the user continuance intention of e-learning. However, adoption of a single model to explain the mHealth continuance use among elders with chronic diseases is limited. Though the ECM-ISC theory can satisfactorily explain the user continuance intention, it ignores the technical factors beyond confirmation and satisfaction. On the contrary, UTAUT is good at analyzing users’ perception of technology and the environmental impact on users. It can effectively measure users’ adoption willingness and user behaviors. The validity of UTAUT is verified by research of different fields, such as building information modeling learning (Peng et al. [28]), online purchasing (Beyari and Garamoun [29]) and micro-lectures (Wijaya and Weinhandl [30]). Though UTAUT is extensively adopted by scholars, this theory ignores the user behavioral intention after adoption. Many scholars hold that comprehensive models can provide more valuable insights in comparison with single models. This conclusion coincides with this research. In this research, ECM-ISC and UTAUT are consequently combined to effectively make up the respective limitations of each model. In order to realize the given research objectives, this research puts forward the following research questions:

Q1: How can mHealth confirmation and satisfaction of elders with chronic diseases influence continuance intention based on the ECM-ISC theory?

Q2: How can effort expectancy, performance expectancy, social influence and facilitating conditions influence mHealth continuance intention of elders with chronic diseases based on the UTAUT?

Q3: Can ECM and UTAUT be effectively combined to form the integrated research model that is applicable to the research field of mHealth?

By addressing these problems, this research can make remarkable contributions to the information system field. Theoretically, UTAUT is introduced on the basis of ECM-ISC in accordance with characteristics of elders with chronic diseases and the mHealth system. The theoretical model that can reflect the mHealth continuance intention of these elders is constructed. SEM is used to verify the validity of the ECM-ISC and UTAUT integrated model in mHealth, which can provide a new perspective for the research of the public health field. In practice, this research can propose development countermeasures and suggestions for mHealth in accordance with research findings. These suggestions can help improve mHealth products, which can promote efficient health management of elders with chronic diseases.

The paper is structured as follows. In the next section, we describe the concept of mHealth, the theoretical background and provide a literature review. Section “Research Model and Hypotheses” reports the proposed research model and hypotheses. Sections “Method” and “Results” reports the instrument development, data collection process, data analysis, and results. In section “Discussion”, we discuss these results. Then, we present the theoretical and practical implications in Section “Implications”. We conclude the paper by summarizing the limitations of the study and suggesting avenues for future research in Section “Limitations and Future Research”.

## 2. Theoretical Background and Literature Review

### 2.1. Mobile Health User Intention

With the acceleration of the global digital health information system, mHealth is an effective way to effectively address patients’ diseases. Wang et al. [31] studied interventions of cancer patients’ health factors and physical activities. Results suggest that mHealth interventions are effective in improving cancer patients’ physical activities and diet. Sawyer et al. [32] research shows that mHealth smoking cessation apps can be feasible and acceptable. Additionally, Mamom and Daovsan [33] evaluate telenursing for caregivers to prevent and treat pressure injury in bedridden patients during the COVID-19 pandemic in Thailand. The research shows that telenursing for caregivers treating and preventing pressure injury in bedridden patients is valuable to the professional consultation during the COVID-19 pandemic. Telenursing can reduce the caregivers’ burden, instructing them how to visually examine, monitor, clean and risk assess the skin of bedridden patients to prevent pressure injury. In addition, mHealth is widely used to explain the decision-making process of mHealth adoption behaviors. For example, Hsiao and Tang [34] pointed out that, based on the TAM (Technology Acceptation Model), the influence of perceived usefulness on mHealth adoption intention of elders in Taiwan is not significant. This research finding shows a good agreement with that of Jen and Hung [35], who also observed an insignificant impact of perceived usefulness on mHealth adoption intention of elders in Taiwan. Conversely, Guo et al. [36] found that perceived usefulness has the most significant influence on Chinese elders’ adoption intention of mHealth services. In the research work of Deng [37], the perceived ease of use of mHealth services is not found with a significant impact on ordinary elders’ user behavior and attitude. Quite the opposite, Hsiao and Tang [34] substantiated that perceived usefulness can significantly influence Chinese elders’ adoption of mHealth services. Additionally, based on the innovation diffusion theory, Hsu et al. [38] listed technological factors, including innovative characteristics, compatibility, complexity, observability, relative advantage and trialability, and verified the impact of the aforesaid technological factors on nurses’ adoption intention of mobile electronic medical services. Following the research work of Hsu et al., Wang and Lin [39] combined the innovation diffusion theory with the task-technology fit model to further verify factors influencing the user intention of big data analytics in mobile cloud health care systems. Additionally, Jiani et al. [40] carried out a survey of mHealth behavioral intention under the background of COVID-19. Results shed light on the fact that the trialability compatibility of mHealth was positively related to the behavioral intention to use. Li [41] conducted a questionnaire of 303 Chinese users, finding out that the attitude toward using mHealth, trust and technology anxiety are significantly associated with users’ behavioral intention to use mHealth. Lee and Fu [42] expanded the UTAUT model for an empirical research of users’ mHealth services in Taiwan. The results reveal that trust, facilitating conditions and performance expectancy have positive effects on satisfaction. Additionally, mHealth knowledge and satisfaction has positive effects on emergency use intention.

On the other hand, many researchers provide solid evidence for the impact of commonly seen factors, such as users’ perceived service risk, quality and environment, on the adoption intention. In terms of perceived service risk, Cocosila and Archer [43] summarized risks influencing users’ adoption intention at the financial, psychological and privacy level. Later, Cocosila [44] added two more factors, including time risk and social risk, to verify the impact of all these factors on the adoption intention. Nisha et al. [45] divided mHealth services at the level of system quality, information quality and interaction quality, and verified how these quality factors indirectly affect the adoption intention via performance expectancy. Chen et al. [46] grouped quality factors into doctor’s service quality and doctor’s information quality and tested how these two quality factors influence users’ continuance intention of mHealth applications via perceived usefulness. Moreover, Ye et al. [7] put forward the resource type and resource accessibility as environmental factors influencing patients’ mHealth usage intention from the perspective of a resource competition perspective. There are also researchers proposing environmental factors, such as legislative protection, based on characteristics of the medical care industry to seek evidence for the impact of these factors on individuals’ adoption of healthcare wearable devices [47].

All the above research findings have provided different perspectives for explanations of mobile medical adoption behaviors. However, research into behavioral intention after adoption is still insufficient. Meanwhile, medical environments, samples and pathology are inconsistent, thus resulting in inconsistency of relevant conclusions. Therefore, this research proceeds from the perspective of elders with chronic diseases to analyze deciding factors influencing mHealth continuance usage intention.

### 2.2. UTAUT (Unified Theory of Acceptance and Usage of Technology)

UTAUT has been the most influential theoretical model following the TAM in the field of information technology adoption. Venkatesh et al. [48] thought that the UTAUT model can explain user adoption and technology use by 70%, which can be an assessment instrument and standard for information technology adoption.

Venkatesh et al. [48] held that a single theoretical model is incomplete in explaining and predicting individual behaviors. Hence, based on the summary of theories related to the TAM, Venkatesh et al. [48] proposed the UTAUT, short for “Unified Theory of Acceptance and Use of Technology”, in response to issues concerning “factors influencing user cognition”. The UTAUT model is actually a combination of eight theoretical models related to information technology adoption and application, including the Technology Acceptance Model, Innovation Diffusion Theory, Task-Technology Fit, Motivational Model, Theory of Reasoned Action, Theory of Planned Behavior, A Model Combined TAM and TPB, Social Cognitive Theory and Model of PC Utilization. It is committed to studying user adoption intention and user behavior of new products and new technologies.

The UTAUT model involves four core variables, chiefly Performance Expectancy (PE), Effort Expectancy (EE), Social Influence (SI) and Facilitating Conditions (FC). Among them, Performance Expectancy (PE), Effort Expectancy (EE), and Social Influence can directly influence user intention, while Facilitating Conditions (FC) has a direct influence on user behaviors.

In fact, the UTAUT, being authoritative and classical, has found wide applications in the information field. For example, Park [49] integrated the UTAUT with the TTF to examine consumer intentions to use a revolutionary technology-driven product. Lu et al. [50] applied the UTAUT model to verify the mobile shopping continuance intention of 866 Chinese consumers and 656 American consumers. Oliveira et al. [51] integrated three models, namely the UTAUT, TTF and ITM, to investigate Portugal users’ behavioral intention and adoption of mobile banking. It can be seen that the UTAUT model has been verified by different fields of information systems. mHealth is an online medical service platform, one kind of information technologies. This research analyzes post-adoption behavioral intention of old patients with chronic diseases. Therefore, we adopt the relatively classical and authoritative UTAUT model as the theoretical basis of this research.

### 2.3. ECM-ISC (Expectation-Confirmation Model of Information System Continuance)

The ECM (Expectation Confirmation Model) was first developed by Oliver [52] as a classical theory to study consumer satisfaction and continuance purchase intention. Because of favorable explanatory power demonstrated by ECM in the field of traditional commerce, some scholars studying information systems started to introduce ECM to the research field of information system. The most representative scholar was Bhattacherjee. Bhattacherjee [25] claimed that users’ repeated use behavior of information system is consistent with the consumer repurchase intention behavior in nature, both of which compare the expected and practical product or service effects to decide whether to repurchase or reuse this product. Therefore, the ECM theory is still applicable to the information system field. Therefore, Bhattacherjee proposed the ECM-ISC theory for the information system based on the ECM theory and the TAM theory in 2001.

Relying on a high prediction accuracy in the information system field, the ECM-ISC theory is widely used to study users’ continuance use intention. For example, Lin et al. [53] studied the continuance intention of web portal users based on the ECM theory, and discovered through empirical survey that perceived playfulness, confirmation to satisfaction and perceived usefulness are all major factors influencing the reuse intention. Lin et al. [54] introduced variables, including perceived privacy risk, perceived enjoyment, perceived reputation and community identification, into the ECM-ISC theory to examine the issue of users’ social networking site continuance. Gu et al. [55] combined the ECM theory with the D&M ISS model, finding that the integrated model can significantly improve the explanatory power of MOOC users’ continuance intention, compared with the original ECM.

A literature review also reveals that the appearance of the ECM and the ECM-ISC has laid a solid theoretical basis for research into consumers’ continuance consumption and continuance use. Nevertheless, this does not mean that these two models are free of any defect. In order to investigate different issues, many scholars have modified the ECM-ISC model, and combined the ECM-ISC with other influencing factors to examine users’ continuance intention. Thereby, this research combines the ECM-ISC and UTAUT to make the research model more consistent with the research topic and research subjects in an attempt to provide a better explanation of mHealth continuance intention of elders with chronic diseases.

## 3. Hypotheses Development and Method

### 3.1. Research Model

**Figure 1:**
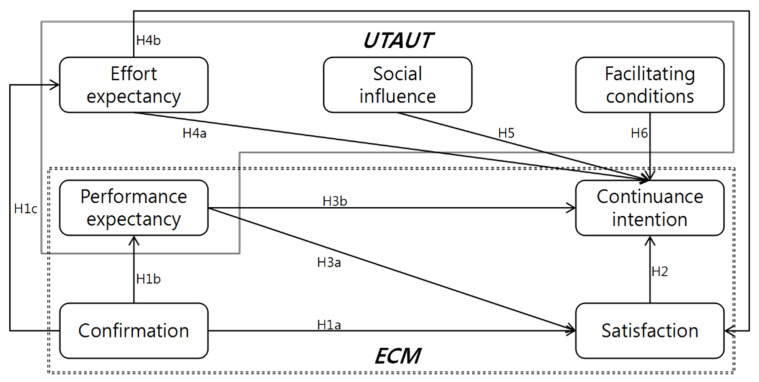
Research model.

According to the extant literature, continuance intention is mostly defined as the behavioral willingness after the first experience of relevant products or services, which can reflect the intensity of users’ willingness to continue using certain information system in the future. Gu [56] believed that initial use is the only first step for the success of an information system, and that to protect users’ continuance intention is actually more critical. So, to explain mHealth continuance intention of elders with chronic diseases, this research establishes a theoretical model based on the ECM-ISC and UTAUT model, which can promote the development of the current mHealth knowledge system. The research model is presented in Figure 1 below:

### 3.2. Hypotheses

In the ECM-ISC, Bhattacheerjee [25] defined how confirmation, perceived usefulness, satisfaction and continuance intention are correlated with each other. Among them, expectation confirmation serves as an independent variable, which can influence perceived usefulness and satisfaction, while perceived usefulness and satisfaction can influence continuance use intention. Moreover, perceived usefulness can also influence satisfaction. Later, some other scholars examined the correlation among confirmation, perceived usefulness and satisfaction in different fields. For example, Oghuma et al. [57] pointed out that perceived usefulness and satisfaction can significantly affect Korean mobile instant messaging users’ continuance intention. In Pang’s [58] research, which was based on the ECM theory, it was pointed out that the expectation confirmation can pronouncedly affect users’ perceived usefulness and satisfaction of knowledge sharing platforms under the background of sharing economy. At the same time, perceived usefulness and satisfaction are also deciding factors of continuance use intention. Additionally, apart from performance expectancy and satisfaction, some research findings have observed that, apart from performance expectancy and satisfaction, confirmation can indirectly influence continuance intention through effort expectancy [59]. Based on the literature review above, the following hypotheses are made:

**Hypothesis** **1a** **(H1a).** *The confirmation has a positive influence on the satisfaction of mHealth’s user.*

**Hypothesis** **1b** **(H1b).** *The confirmation has a positive influence on the performance expectancy of mHealth’s user.*

**Hypothesis** **1c** **(H1c).** *The confirmation has a positive influence on the effort expectancy of mHealth’s user.*

**Hypothesis** **2** **(H2).** *The satisfaction has a positive influence on the continuance intention of mHealth’s user.*

Similar to perceived usefulness in TAM, performance expectancy refers to individual perception of to what extent a system is helpful to his work [48]. In this research, performance expectancy is defined as a subjective perception of patients with chronic diseases about how use of mHealth can improve or help promote their health. Perceived usefulness is the linchpin to users’ adoption of information technology. If elders with chronic diseases perceive that mHealth can improve their health management efficiency, their use intention will be stronger. On the contrary, when they perceive that system use will benefit them in no way, their user intention will be weakened. Research of Riad et al. [60] suggests that performance expectancy is a primary factor affecting users’ intention to use the mHealth information system. More than that, Wu and Tian [61] found that performance expectancy can not only significantly influence user satisfaction of the information system, but also affect the most significant influencing factors of continuance use intention. On account of what is discussed above, the following hypotheses are made:

**Hypothesis** **3a** **(H3a).** *The performance expectancy has a positive influence on the satisfaction of mHealth’s user.*

**Hypothesis** **3b** **(H3b).** *The performance expectancy has a positive influence on the continuance intention of mHealth’s user.*

Effort expectancy is similar to the perceived ease of use in the TAM, which can be regarded as how much effort a person makes to use a system [48]. In this research, it is defined by the perceived ease of use of elders with chronic disease about mHealth. Previous research suggests that effort expectancy can significantly influence users’ adoption of the health information system [62,63]. Because of physiological and psychological characteristics, elders with chronic diseases might feel stressful about using mHealth. If mHealth is easy to use, elders with chronic diseases will be satisfied with mHealth and willing to continue using it. In contrast, if it is very difficult to use, elders will feel dissatisfied with their user experience, and their continuance intention will be correspondingly weakened. So, the following hypotheses are made:

**Hypothesis** **4a** **(H4a).** *The effort expectancy has a positive influence on the satisfaction of mHealth’s user.*

**Hypothesis** **4b** **(H4b).** *The effort expectancy has a positive influence on the continuance intention of mHealth’s user.*

Social influence means the degree to which an individual is influenced by the surrounding crowd in using a system [48]. This research defines social influence as to which degree an individual is influenced by people around him, thus being willing to continue using an information system. Bandura [64] held that social influence plays a critical role in human behaviors and decision-making. Additionally, Dwivedi et al. [65] found that an individual tends to adjust his attitude according to others’ information and stories. Lu et al. [66] observed that social influence has a significant impact on users’ adoption intention of an information system. In Sun’s research [62], it was also pointed out that social influence is a major influencing factor of users’ mHealth adoption.

Inevitably, use of mHealth by elders with chronic diseases will be influenced by their family members, friends or other people around them. When others support, encourage or recommend use of mHealth, elders will be more willing to use it. This means a positive attitude of others towards mHealth can improve continuance intention of elders with chronic diseases. If others are adverse towards or even reject use of mHealth, mHealth continuance intention of elders with chronic disease will be influenced. Hence, the following hypotheses are made:

**Hypothesis** **5** **(H5).** *The social influence has a positive influence on the continuance intention of mHealth’s user.*

Facilitating conditions refer to an individual’s perception of organization’s support for system use in terms of relevant technologies and facilities [48]. In this research, facilitating conditions are defined as an individual’s perception of mHealth service support and technological support can facilitate his continuance use of mHealth. Yi et al. [67] claimed that facilitating conditions can directly decide information system adoption. Bhattacherjee [68] also provided solid evidence for the critical role of infrastructure support in health information adoption.

mHealth can be used on the mobile phone or on the tablet PC. If an individual has devices which can support the use of mHealth, it can promote the user behavior. Additioanlly, the prerequisite for elders’ use of an information system is usually service support. Only when elders with chronic diseases can receive timely service support for their puzzlements in using the mHealth system, they will be willing to continue using the mHealth system. Hence, the following hypothesis is made:

**Hypothesis** **6** **(H6).** *The facilitating conditions has a positive influence on the continuance intention of mHealth’s user.*

### 3.3. Method

The structural equation modeling (SEM) is a technique that combines statistics and qualitative causality hypothesis to assess the cause-and-effect correlation. The SEM analysis simultaneously considers and copes with multiple dependent variables. In regression analysis or path analysis, even if multiple dependent variables are displayed in graphs of statistical results, the dependent variables are still computed one by one to work out the regression coefficient or path coefficient. In addition, variables such as attitude and behavior usually contain errors, which cannot be measured by single indexes. The SEM analysis allows independent variables and dependent variables to contain measurement errors. Consequently, in order to measure the behavioral willingness of elders with chronic diseases to continue using mobile medical devices, the data analysis of this research is completed through SEM.

The data were searched from online and offline channels. Finally, 1453 copies of the valid questionnaire were collected. In order to guarantee validity of the questionnaire, the valid copies of the questionnaire were screened out, with 117 copies of questionnaire that were invalid and dishonest deleted. In order to ensure the validity and integrity of the questionnaire, we first eliminated those with questions unfilled. We defined questionnaires with 80% of the questions answered with the same number as invalid samples. Questionnaires with an obvious answering rule (such as 1, 2, 3, 4… 1, 2, 3, 4…) were also deemed as invalid. As to online copies, we invited elders with chronic diseases and mHealth user experience through doctors or nurses of medical institutions to fill in the questionnaire, finally collecting 926 valid copies. As to offline copies, we visited large-scale medical institutions in person to invite elders with chronic diseases and mHealth user experience to fill in the questionnaire, finally collecting 527 copies. The whole survey continued for two months. No significant differences between respondents of the first month and the second month were found. Finally, the data obtained from the questionnaire are analyzed below using IBM SPSS v23.0 and IBM AMOS v23.0 (IBM, Armonk, NY, USA).

All questions were statistically analyzed by the 7-point Likert scale, where “1” indicates “strongly disagree” and “7” indicates “strongly agree”. Appendix A presents the final questionnaire.

Table 1 lists the demographic statistics of research samples. As one can observe, there are 891 males and 562 females, taking up 61.3% and 38.7% of the total, respectively. All the research samples are aged above 60 years old. As to their educational background, those graduating from junior colleges are in the highest percentage, as high as around 38.5%. Respondents using mHealth for more than one year are around 587, taking up 40.4% of the total.

## 4. Results

### 4.1. Measurement Model

First, we conducted a reliability and convergent validity. SPSS (IBM, Armonk, NY, USA) was used to carry out reliability analysis of the questionnaire. Cronbach’s α of every variable is above 0.8, which exceeds the general level, 0.7. This suggests favorable reliability of the measurement model [69]. AMOS (IBM, Armonk, NY, USA) was used to conduct a convergent validity test of the questionnaire. All loadings are higher than 0.4, and the composite reliability (CR) of every variable is higher than 0.6. Meanwhile, the average variance extracted (AVE) of every variable is larger than 0.5, suggesting favorable validity of the measurement model.

Table 2 lists the standardized item loadings, the CR (Composite reliability), the AVE (Average variance extracted) and Cronbach Alpha values. As listed in the table, most item loadings are larger than 0.7. Each AVE exceeds 0.5, and each CR exceeds 0.8. In addition, all Cronbach Alpha values are larger than 0.8. This indicated the excellent convergent validity and reliability [69].

Discrimination validity refers to the low correlation and significant difference between latent variables, and it can be evaluated by comparing AVE square root and correlation coefficients between variables. If the correlation coefficient of a variable with another one is smaller than the square root of the average variance of the variable, it indicates that the discrimination validity of the variable is good [69]. 

Table 3 is a summary of correlation coefficients among seven latent variables. The number on the diagonal line is the square root (in bold numbers) of every variable’s AVE. The square root of AVE of every variable falls between 0.752 and 0.811. The absolute value of the correlation efficient between different variables is smaller than 0.7. The square root of the AVE of every variable is obviously larger than the correlation coefficient between the variable and other variables. This suggests favorable discriminant validity among the 7 latent variables.

Further, as presented by Henseler et al. [70], we tested the heterotrait–monotrait ratio (HTMT). If the HTMT value is below 0.85, discriminant validity is established between two reflective constructs. Table 4 shows the result of HTMT against our data. All the values meet the threshold.

### 4.2. Structural Model

We adopted structural equation modeling software AOMS 23.0 (Sharp Shape, Saratoga, CA, USA) to estimate the structural model. Table 5 lists the recommended value [69] and actual values of structural model fit: all fit indices have better actual values than the recommended values.

**Figure 2:**
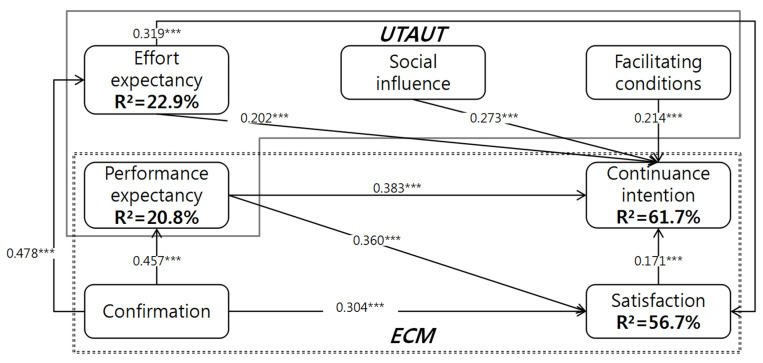
Structural model results. Note: *** *p* < 0.001; the dotted line represents the insignificant path.

Figure 2 and Table 6 show the AMOS 23.0 estimation results. The model explains 61.7% of continuance intention to use mHealth. Hypotheses related to continuance intention H2, H3b, H4, H5 and H6 are confirmed. The model explains 20.8% of variation in performance expectancy, and validates the Hypothesis (H1b). This model explains 56.7% of the variation in satisfaction, and confirms hypotheses between the determinants satisfaction, performance expectancy, effort expectancy and confirmation (H1a, H3a, H4b). This model explains 22.9% of the variation in effort expectancy. The results also confirm the hypotheses between effort expectancy and confirmation (H1c). The analysis results can be summarized as follows.

The performance expectancy was statistically significant in explaining the confirmation (β = 0.457; *p* < 0.01), thus confirming Hypothesis H1b. The results indicate that confirmation was the most important construct in explaining the performance expectancy in mHealth. In other words, when confirmation increased one standardized unit, performance expectancy increased 0.457 standardized units, ceteris paribus. The model explains 20.8% of the variation in the performance expectancy.

The effort expectancy was statistically significant in explaining the confirmation (β = 0.478; *p* < 0.01), thus confirming Hypothesis H1c. The results indicate that confirmation was the most important constructs in explaining the effort expectancy in the mHealth. In other words, when confirmation increased one standardized unit, effort expectancy increased 0.478 standardized units, ceteris paribus. The model explains 22.9% of the variation in the effort expectancy.

The confirmation (β = 0.304; *p* < 0.01), performance expectancy (β = 0.360, *p* < 0.01) and effort expectancy (β = 0.319; *p* < 0.01) were statistically significant in explaining satisfaction, thus confirming Hypotheses H1a, H3a and H4a. The results indicate that performance expectancy was the most important construct to explain the satisfaction given that, when the performance expectancy increased one standardized unit, satisfaction increased 0.360 standardized units, ceteris paribus. The model explains 52.5% of the variation in the satisfaction of mHealth.

The satisfaction (β = 0.171; *p* < 0.01), performance expectancy (β = 0.383, *p* < 0.01), effort expectancy (β = 0.202; *p* < 0.01), social influence (β = 0.273; *p* < 0.01) and facilitating conditions (β = 0.214; *p* < 0.01) were statistically significant in explaining continuance intention, thus confirming Hypotheses H2, H3b, H4a, H5 and H6. The results indicate that performance expectancy was the most important construct to explain the continuance intention given that, when the performance expectancy increased one standardized unit, continuance intention increased 0.383 standardized units, ceteris paribus. The model explains 61.7% of the variation in the continuance intention of mHealth.

## 5. Discussion and Conclusions

### 5.1. Discussion

The purpose of this research is to examine factors influencing mHealth continuance use of elders with chronic diseases. An integrated model is constructed on the basis of the ECM-ISC and UTAUT. In addition, ten hypotheses stated above are analyzed based on the structural equation model. Analysis results indicate that the ten hypotheses made by this research are all substantiated.

First of all, confirmation can significantly influence satisfaction (β = 0.304; *p* < 0.01); performance expectancy (β = 0.457; *p* < 0.01); and effort expectancy (β = 0.478; *p* < 0.01). These results are consistent with those of previous research findings [57,59]. Since confirmation involves comparison of user satisfaction before and after use, when initial use by elders with chronic diseases can satisfy the individual’s psychological expectation, it can effectively promote mHealth user satisfaction, performance expectancy and effort expectancy of elders with chronic diseases.

Additionally, performance expectancy (β = 0.304; *p* < 0.01), performance expectancy (β = 0.457; *p* < 0.01) and satisfaction (β = 0.171; *p* < 0.01) can significantly influence continuance intention. On the other hand, satisfaction is significantly influenced by performance expectancy (β = 0.360; *p* < 0.01) and effort expectancy (β = 0.319; *p* < 0.01). Among them, the influence of performance expectancy on continuance intention is the most significant. Results suggest that the more helpful mHealth is to health management efficiency and facilitating conditions of elders with chronic diseases, the more satisfied they will be and the stronger their mHealth continuance intention will be. This fully indicates that, if mHealth is better than traditional offline medical care models, enabling elders with chronic diseases to more conveniently improve their health management performance, then they might have a stronger mHealth continuance intention. These results are consistent with those of previous research findings [55,61].

In the end, social influence (β = 0.273; *p* < 0.01) and facilitating conditions (β = 0.214; *p* < 0.01) can significantly influence continuance intention. These results are consistent with those of previous research findings as well as hypotheses of the original UTAUT [71,72]. First of all, elders with chronic diseases are living in a social environment, so the social influence on elders cannot be ignored. Currently, the health status of a majority of elders is not optimistic. Elders have increased their communication about diseases with each other. If these groups can encourage patients with chronic diseases to use mHealth for self-health management, patients with chronic diseases will have a positive attitude towards mHealth, which can promote mHealth continuance use. On the other hand, many patients with chronic diseases rely on mHealth for real-time monitoring of their personal health. This means that if infrastructure, such as WiFi, is complete it can also influence elders’ mHealth continuance intention. Moreover, digital life can be a great puzzlement to elders so whether there are consulting services and timely solutions to elders’ puzzlement over use of mHealth is also a major factor influencing whether elders with chronic diseases will continue using mHealth.

### 5.2. Implications

mHealth cannot give full play to its value only when it is used by patients with chronic diseases in the long term. Previous research focused on the functional design and advantages of the health management system. On the contrary, this research proceeds from the perspective of elders with chronic diseases to examine factors influencing mHealth continuance intention. Researchers verify the validity of ECM-ISC and UTAUT integrated model in the field of mHealth. This can provide other researchers with new critical evidence. We suggest that future researchers should continue their exploration of the mHealth user adoption based on these two theories. Additionally, research findings of this research can give some implications to management personnel. Proceeding from influencing factors, this research proposes countermeasures for further improvement of mHealth so as to deepen the mHealth continuance intention among elders with chronic diseases and give fuller play to the value of mHealth among elders and patients with chronic diseases.

From the theoretical perspective, ECM-ISC and UTAUT are both relatively authoritative and classical theories in the field of information system, which have been extensively verified by numerous scholars. Nevertheless, each model has its limits. ECM-ISC, though capable of favorably explaining users’ continuance intention, ignores external factors influencing user expectations. UTAUT is a good choice to examine user psychological traits and attitudes and can well measure users’ initial adoption intention and user behavior, but it ignores user behavioral intention after use. Therefore, in order to better explain users’ continuance intention and increase the consistency of the research model with the research topic, this research integrates ECM-ISC with UTAUT to explain mHealth continuance intention of elders with chronic diseases. Compared with ECM-ISC or UTAUT alone, the integrated model can provide more explanations for continuance intention. The integrated model can explain 61.7% of the variation in continuance intention, which is significantly higher than 41% by the original ECM-ISC. This can promote development of continuance intention research, and even hold vital academic significance to information system adoption. In the future, these two kinds of opinions can be combined to study user adoption of other information system fields. We think that the integrated model can offer more insights, compared with the single research model or perspective.

This research is helpful to mHealth providers’ formulation of effective strategies for the improvement of user demand and user participation. First of all, confirmation can significantly influence satisfaction, performance expectancy and effort expectancy. Performance expectancy and effort expectancy can directly and significantly influence continuance intention, and can indirectly influence continuance intention via satisfaction. Among them, the direct influence of performance expectancy on continuance intention is the most significant. Therefore, mHealth providers, while developing mHealth functions, should consider expectations of elders with chronic diseases about mHealth functions or services. Elders can even be invited to give some suggestions on product improvement so that mHealth functions can effectively improve their health management performance. Additionally, mHealth should spare no effort to deepen elders’ understanding of mHealth’s competitive advantages over other health management information systems in functions. So, emphasis should be laid on highlighting facilitating conditions of the information system design. The system interface should be concise; texts, images and videos should be well matched and distributed to reduce information overloading.

Other than that, social influence can significantly influence continuance intention. Elders with chronic diseases can easily change their user behaviors and attitudes because of others’ user habits, comments and opinions. Therefore, mHealth can enhance its own publicity and promotion. On the other hand, it can make use of multi-channel socializing platforms to periodically launch different topics and activities to attract user attention, enhance interaction with users and shorten the distance between users and operators. Moreover, mHealth can seek collaboration with Internet celebrities or stars to develop opinion leaders. These opinion leaders can foster and strengthen mHealth continuance intention of elders with chronic diseases. Additionally, facilitating conditions can significantly influence elders’ continuance intention. So, mHealth should not only provide detailed manual instructions for elders to accelerate their familiarity with mHealth operations, but also provide timely consulting services or help for elders when they have difficulty using mHealth.

Therefore, efforts should be made to build an effective doctor and health management plan assessment mechanism, in addition to providing health management files and health management information. The newly established mechanism should be able to assess doctors and health management plans as well as doctors’ personalized characteristics. This can give potential users references on which health management plan or doctor to choose. Other than that, adequate attention should be paid to the expectations of critical prerequisites which can influence the user continuance intention. It is suggested that mHealth operators should highlight their practicability, maintain a close connection with users, and immediately learn users’ expectations and demands of health management and system. Additionally, they should honestly admit the practical performance and limitations of mHealth, avoiding exaggerating the advertised functions of mHealth, because the impractical initial expectations caused by exaggerating advertising are not beneficial to the formation of serviceability and satisfaction. Thereby, the user continuance intention will be impaired.

### 5.3. Limitations and Future Research

This research revolves around mHealth continuance intention of elders with chronic diseases. In spite of satisfactory reliability and validity achieved by the theoretical model constructed hereunder, this research cannot avoid the following limitations because of limited human power and material resources. First of all, we explain mHealth continuance intention of elders with chronic diseases from the combined perspective of the ECM-ISC and UTAUT. Future researchers can take into account more influencing factors, such as perceived value, trust and privacy, to further improve the scientificity and feasibility of the research model.

Second, all respondents of this research are Chinese. Our research results might not be applicable to other countries. So, the feasibility of our research model to other countries or regions is calling for further verification.

At last, all respondents are elders with chronic diseases. In the future, patients with acute diseases can be included to examine the differences between continuance intention of patients with acute diseases and chronic diseases.

### 5.4. Conclusions

As an alternative plan of traditional medical care, mHealth has gained increasing attention worldwide from governments to individuals. However, there is not yet any research attempt comprehensively assessing the mHealth continuance intention from the perspective of elders with chronic diseases. In order to make up the research gap, we integrated the two authoritative theories, namely ECM-ISC and UTAUT, to draw up an innovative research model. Data were gathered from Chinese respondents to test the integrated research model and to thus identify major antecedents of the continuance intention. This can effectively make up the research gap of mHealth. It was also the first time that the user continuance intention was tested among elders with chronic diseases. Our research results suggests that the integrated research model possesses favorable explaining power. This can help substantiate the validity of the integrated research model in analyzing the mHealth continuance use intention among these patients. Results reveal that satisfaction, performance expectancy, effort expectancy, social influence and facilitating conditions have a direct influence on the mHealth continuance intention, and that confirmation could play a role through satisfaction, performance expectancy and effort expectancy. This research can provide a solid foundation for the improvement of the continuance intention model. Meanwhile, findings of this research are also critical to mHealth operators’ understanding of user expectations of mHealth service design, improvement and implementation. All these services and functions can stimulate the mHealth continuance intention among elders with chronic diseases and crying for long-term health management.

## Figures and Tables

**Table 1:** Sample characteristics.

Variable		Number	Percentage
Gender	Male	891	61.3%
	Female	562	38.7%
Age	60–65	792	54.5%
	66–70	474	32.7%
	71–75	159	10.9%
	>75	28	1.9%
Education	Middle school or below	52	3.6%
	Senior high school	437	30.1%
	Junior college	559	38.5%
	Undergraduate	371	25.5%
	Postgraduate or above	34	2.3%
Usage history	<1 month	441	30.4%
	1–6 months	232	15.9%
	6–12 months	193	13.3%
	>1 year	587	40.4%
Experience	Yes	1453	100%

**Table 2:** Standardized item loadings, CR, AVE and alpha values.

Factor	Item	Standardized Loadings	Alpha	AVE	CR
Effort expectancy (EE)	EE1	0.805	0.848	0.590	0.852
	EE2	0.805			
	EE3	0.726			
	EE4	0.733			
Social influence (SI)	SI1	0.795	0.858	0.602	0.858
	SI2	0.781			
	SI3	0.752			
	SI4	0.773			
Facilitating conditions (FC)	FC1	0.723	0.857	0.601	0.857
	FC2	0.796			
	FC3	0.775			
	FC4	0.803			
Performance expectancy (PE)	PE1	0.741	0.882	0.658	0.885
	PE2	0.735			
	PE3	0.763			
	PE4	0.766			
Confirmation (Con)	Con1	0.790	0.870	0.626	0.870
	Con2	0.792			
	Con3	0.813			
	Con4	0.848			
Satisfaction (Sat)	Sat1	0.773	0.838	0.565	0.838
	Sat2	0.801			
	Sat3	0.794			
	Sat4	0.795			
Continuance intention	CI1	0.803	0.869	0.628	0.871
	CI2	0.743			
	CI3	0.766			
	CI4	0.853			
	EE1	0.805			
	EE2	0.805			

**Table 3:** Matrix of correlation constructs and discriminant validity.

	EE	SI	FC	Con	Sat	PE	CI
**EE**	**0.768**						
**SI**	0.361	**0.776**					
**FC**	0.297	0.263	**0.775**				
**Con**	0.468	0.251	0.290	**0.811**			
**Sat**	0.583	0.356	0.351	0.605	**0.791**		
**PE**	0.380	0.307	0.295	0.462	0.603	**0.752**	
**CI**	0.564	0.534	0.482	0.269	0.657	0.666	**0.792**

Note: The square root of AVE (shown as bold at diagonal) and factor correlation coefficients.

**Table 4:** Heterotrait-Monotrait Ratio (HTMT).

	CI	Con	EE	FC	PE	SI	Sat
**CI**							
**Con**	0.277						
**EE**	0.576	0.480					
**FC**	0.485	0.296	0.301				
**PE**	0.672	0.472	0.381	0.299			
**SI**	0.542	0.253	0.366	0.264	0.306		
**Sat**	0.658	0.615	0.587	0.353	0.603	0.354	

**Table 5:** Fit indicators of the structural models.

Model Fit Indices	χ^2^/DF	AGFI	RMSEA	IFI	GFI	CFI	NFI
Recommended value	1–3	>0.80	>0.05	>0.90	>0.90	>0.90	<0.90
Actual value	2.846	0.946	0.036	0.971	0.955	0.971	0.956

Notes: χ2/d.f. chi-squared divided by degrees of freedom; AGFI, adjusted goodness-of-fit index; RMSEA, root mean square error of approximation; IFI, incremental fit index; GFI, goodness-of-fit index; CFI, comparative fit index; NFI, normed fit index.

**Table 6:** Results of the hypotheses tests.

Path		Estimate	S.E.	*p*-Value (C.R.)	Results
H1a	Confirmation→Satisfaction	0.304	0.029	(9.843) ***	Supported
H1b	Confirmation→Performance expectancy	0.457	0.027	(14.576) ***	Supported
H1c	Confirmation→Effort expectancy	0.478	0.030	(15.736) ***	Supported
H2	Satisfaction→Continuance intention	0.171	0.035	(4.812) ***	Supported
H3a	Performance expectancy→Satisfaction	0.360	0.032	(12.422) ***	Supported
H3b	Performance expectancy→Continuance intention	0.383	0.035	(12.046) ***	Supported
H4a	Effort expectancy→Continuance intention	0.202	0.027	(7.048) ***	Supported
H4b	Effort expectancy→Satisfaction	0.319	0.027	(11.293) ***	Supported
H5	Social influence→Continuance intention	0.273	0.023	(11.199) ***	Supported
H6	Facilitating conditions→Continuance intention	0.214	0.026	(8.828) ***	Supported

Note: *** *p* < 0.001.

## Data Availability

Not applicable.

## Appendix A. The Study’s Measurement Items

**Dimensions**	**Texts of Items**
Effort expectancy	Learning how to use mHealth is easy for me.
	My interaction with mHealth is clear and understandable.
	I find mHealth easy to use.
	It is easy for me to become skilful at using mHealth.
Social influence	People who are important to me think that I should use mHealth.
	People who influence my behaviour think that I should use mHealth.
	People whose opinions that I value prefer that I use mHealth.
	Use of mHealth gives me social status.
Facilitating conditions	I have the resources necessary to use mHealth.
	I have the knowledge necessary to use mHealth.
	mHealth are compatible with other technologies I use.
	I can get help from others when I have difficulties using mHealth.
Performance expectancy	I find mHealth useful in my daily health management.
	Using mHealth increases my health that are important to me.
	Using mHealth helps me accomplish health management more quickly.
	Using mHealth increases my health management productivity.
Confirmation	My experience with using the mHealth was better than I expected.
	The service level provided by the mHealth was better than I expected.
	The service level or function provided for mHealth in general was better than I predicted.
	Overall, most of my expectations from using mHealth were confirmed.
Satisfaction	I feel satisfied with using mHealth.
	I feel contented with using mHealth.
	I feel pleased with using mHealth.
	I believe I made the correct decision in using a mHealth.
Continuance intention	I intend to continue using mHealth in the future.
	I will continue using the mHealth in future.
	I will maintain my mHealth use frequency in the future.
	I will recommend the mHealth to others.
